# Informing Balanced Investment in Services and Health Systems: A Case Study of Priority Setting for Tuberculosis Interventions in South Africa

**DOI:** 10.1016/j.jval.2020.05.021

**Published:** 2020-11

**Authors:** Fiammetta M. Bozzani, Tom Sumner, Don Mudzengi, Gabriela B. Gomez, Richard White, Anna Vassall

**Affiliations:** 1 Department of Global Health and Development, London School of Hygiene and Tropical Medicine, London, England, UK; 2 TB Modelling Group, Department of Infectious Disease Epidemiology, London School of Hygiene & Tropical Medicine, London, England, UK; 3 The Aurum Institute, Johannesburg, South Africa; 4 Sanofi Pasteur SA, Vaccine Epidemiology and Modelling, Lyon, France

**Keywords:** health system constraints, model-based economic evaluation, priority setting

## Abstract

**Objectives:**

Health systems face nonfinancial constraints that can influence the opportunity cost of interventions. Empirical methods to explore their impact, however, are underdeveloped. We develop a conceptual framework for defining health system constraints and empirical estimation methods that rely on routine data. We then present an empirical approach for incorporating nonfinancial constraints in cost-effectiveness models of health benefit packages for the health sector.

**Methods:**

We illustrate the application of this approach through a case study of defining a package of services for tuberculosis case-finding in South Africa. An economic model combining transmission model outputs with unit costs was developed to examine the cost-effectiveness of alternative screening and diagnostic algorithms. Constraints were operationalized as restrictions on achievable coverage based on: (1) financial resources; (2) human resources; and (3) policy constraints around diagnostics purchasing. Cost-effectiveness of the interventions was assessed under one “unconstrained” and several “constrained” scenarios. For the unconstrained scenario, incremental cost-effectiveness ratios were estimated with and without the costs of “relaxing” constraints.

**Results:**

We find substantial differences in incremental cost-effectiveness ratios across scenarios, leading to variations in the decision rules for prioritizing interventions. In constrained scenarios, the limiting factor for most interventions was not financial, but rather the availability of human resources.

**Conclusions:**

We find that optimal prioritization among different tuberculosis control strategies in South Africa is influenced by whether and how constraints are taken into consideration. We thus demonstrate both the importance and feasibility of considering nonfinancial constraints in health sector resource allocation models.

## Highlights

• Health system constraints such as financial, human resources, and diagnostic inputs scarcity can influence the opportunity costs of health interventions and prevent the efficient allocation of resources. Methods for empirical estimation and inclusion of these constraints in priority setting are underdeveloped, particularly for application in low- and middle-income settings.
• We propose an approach that can be applied using routine data and that, crucially, presents decision makers with a choice set including constrained and unconstrained scale-up options as well as, crucially, the option of relaxing the health system constraints. The approach is illustrated using a case study of developing an essential tuberculosis control package in South Africa.
• We find that taking health system constraints into account changes the cost-effectiveness ranking of intervention options, demonstrating their importance for priority setting. We demonstrate that the approach is feasible within a policy-driven timeline.

## Introduction

Health sectors face nonfinancial constraints that prevent the efficient allocation of resources. Nonfinancial constraints have consequences for the assessment of cost-effectiveness because they can influence the opportunity cost of new interventions and technologies.^1^^,^^2^ Supply-side (or health systems) nonfinancial constraints occur when factors of production (inputs) are “fixed” in the short run, either owing to physical and external barriers (for example, it may take 5 years to train health professionals, there may be political barriers to immigration, or “sunk” costs in operating theaters may prevent use of funds to expand outpatient facilities), or owing to health sector actors deliberately constraining resource availability/flexibility through policy decisions (for example, accreditation systems restricting the supply of clinical labor, or budgeting and procurement practices). Whatever their nature, nonfinancial constraints ultimately impact the health sector’s ability to react to technological change by “fixing” the levels and use of specific sets of inputs, and consequently, in the short run, new technologies dependent on these inputs may have higher opportunity costs than they would if all inputs were variable.

Van Baal and colleagues have previously presented both a theoretical and an empirical approach for estimating the extent to which nonfinancial constraints affect opportunity costs.^1^ They characterize the decision under two separate constraints: one for the general budget and one for the constrained input; they recognize that the constrained input has a lower cost-effectiveness threshold (*k*_*1*_) than the unconstrained input (*k*_*0*_), which reflects its higher opportunity cost, so that *k*_*1*_ < *k*_*0*_. The traditional decision rule, comparing the incremental cost per unit of health produced by the intervention to the general opportunity cost threshold, can thus be modified by adjusting the opportunity cost of the constrained input by the ratio $\frac{{\text{k}}_{0}}{k_{1}}$.

Empirically, van Baal and colleagues posit that the relative costs of constrained to nonconstrained inputs per unit of outcome in the current standard of care reflect their relative current opportunity cost. Assuming constant returns to scale and perfectly divisible inputs, they provide a 2-intervention, 2-input model:

(1) $$\frac{k_{0}}{k_{1}}=\frac{s-t}{q-p}$$

where *s* and *t* denote the unit costs of interventions *i* and *j*, while *q* and *p* denote the costs of the constrained inputs for the same interventions, respectively.^1^

This approach can be used to empirically correct for downward bias in incremental cost-effectiveness ratios (ICERs) of new technologies implemented in health systems where specific inputs are constrained (or upward bias in rarer cases of existing spare capacity in the system), and where data are available on both the cost of the intervention and standard of care divided into constrained and unconstrained inputs. In principle, this approach could also be extended to within health sector budget constraints, for example, when dealing with fixed budgets for different disease programs. In this case, groups of disease-specific inputs have a higher opportunity cost than spending on other areas of the health system. Potentially, once the comparative cost per health outcome is known for each program, the incremental cost-effectiveness of the program may be adjusted using the same approach.

Based on an application by Revill and colleagues, this approach appears empirically feasible.^3^ It has several limitations, however, that can be grouped into 2 sets: one that restricts its applicability to certain settings and another that questions its underlying assumptions. First, in many low- and middle-income countries (LMICs) there remains a substantial empirical challenge in deriving cost estimates. Given data scarcity on the relationship between costs, outputs, and outcomes, it is unlikely that decision makers are sufficiently informed for costs to represent current relative opportunity costs of inputs. Another setting-specific limitation is that the approach relies on the estimation of health sector opportunity cost-based thresholds, which add substantial uncertainty. With regard to the more general limitations, the assumption of constant returns to scale for inputs is only likely to hold for some inputs. Moreover, as it stands, the approach proposed by van Baal and colleagues and its subsequent application do not present the decision maker with a choice set that includes relaxing nonfinancial constraints.

### Empirical Approaches

Decision makers prioritizing new technologies (or packages of interventions across specific disease areas) often do not have control over decisions made around the wider health system constraints. For example, the manager of a human immunodeficiency virus (HIV) program may be able to decide to invest in a new HIV treatment but may not be able to determine the overall level of nurses available in the health sector. Ideally, when the supply of nurses is constrained, the HIV program would prioritize interventions with a lower demand on nurses than it would in the absence of constraints. Alternatively, program managers may consider cofinancing interventions that relax the overall nursing constraints, although this rarely happens in practice.^4^

Methods to support priority setting for investment in infectious disease programs are evolving and receiving increased academic attention. The widespread use of mathematical modeling in economic evaluation for infectious disease interventions has enabled optimization among multiple interacting intervention options under a disease-specific budget constraint.^5^^,^^6^ Currently, these models estimate the costs of interventions and comparators by multiplying services produced by their average cost or, in some cases, employing a cost function.^7^^,^^8^ We present an empirical method that builds on the strengths of these models. Our approach has 4 stages: (1) consulting decision makers to elicit nonfinancial constraints applicable to the specific setting and interventions of interest; (2) quantifying the constraints and their impact; (3) employing economic evaluation models (in this case mathematical models) to estimate costs and quantities of constrained inputs; (4) producing ICERs both with and without constraints. We illustrate the importance and feasibility of our method through a case study, conducted using secondary and routine data within a policy-led (national strategic planning) time frame.

## Methods

### Case Study Setting and Interventions

Tuberculosis (TB) control is a major concern for the South African health system.^9^ In 2015, plans were announced for a comprehensive TB screening program, a component of which involves using intensified case finding (ICF) to screen every person attending a public health facility for any reason. Initial (conventional) mathematical modeling of the potential impact found that scale-up of ICF to all health facility attendees is the single most effective intervention for reaching the post-2015 global TB targets,^10^ but is also the costliest screening option.^11^ Policy makers in South Africa, however, were concerned about the feasibility of implementing such a complex intervention at full scale in a health system that they felt was “overstretched.” As part of the National Strategic Plan (2017-2022) priority-setting process, we therefore reassessed ICF policy options incorporating supply-side health system constraints, adding a measure of the feasibility of these options in terms of the costs of relieving the constraints.

Strategies to identify and treat people with TB, especially those who have not sought diagnostic services on their own initiative, have the potential to become an integral part of TB control in LMICs, where access to health services is poor, even among symptomatic patients, and there is limited capacity among health providers to recognize symptoms.^12^ ICF, or facility-based screening, was adopted in South Africa for detecting TB cases among HIV clinic attendees, who are screened for TB at each visit. The TB program, though, still relies on “passive case-finding,” screening only those individuals presenting with symptoms suggestive of TB for identifying cases among those who are HIV uninfected. This method was shown to miss a large proportion of facility-based TB cases presenting at health facilities for reasons other than respiratory symptoms.^13^ TB symptom screening traditionally relies on triaging patients based on the presence of prolonged cough. The World Health Organization (WHO) recently developed a more sensitive but less specific screening tool based on the presence of any of 4 symptoms (current cough, fever, weight loss, or night sweats) for use among patients with HIV in LMICs, which showed potential as part of a clinical scoring system for prioritizing TB investigation among symptomatic individuals in South Africa.^14^

Once referred for TB testing, suspects should go on to be tested with Xpert MTB/RIF, which was rolled out in South Africa to replace sputum smear microscopy in 2012 and reached an estimated coverage of 80% in 2016.^15^ Those patients who are HIV infected (or whose HIV status is unknown) and who receive a negative Xpert result should then give a second sputum sample investigated using TB culture, although adherence to the follow-up test is poor.^16^

**Figure 1:**
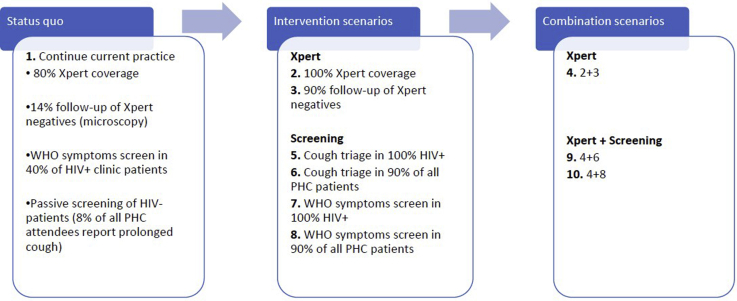
Modeled interventions.

The sensitivity and specificity of the chosen screening and diagnostic algorithm determine the consequential costs of diagnosis and treatment along the TB care cascade and are therefore a crucial consideration for priority-setting in a resource-constrained health system. For this reason, the South African TB Think Tank, which supports TB policy making, was tasked with carrying out a model-based economic evaluation to prioritize among the alternative algorithms considered for inclusion in the latest National Tuberculosis Plan (NTP). The status quo and intervention scenarios considered in the analysis and the respective policy-defined coverage targets are described in Figure 1.

### Identifying Constraints Scenarios

Our approach was embedded within the strategic planning cycle through the South African TB Think Tank, a body that reviews evidence on TB interventions and was responsible for recommending those to be considered in the NTP.^17^ The TB Think Tank were asked to identify the main constraints on TB case detection. The process of elicitation and quantification of the constraints has been described in detail elsewhere.^18^ The selection process relied on a published framework^2^ and aimed to illustrate the different forms that constraints might take in influencing the priority-setting process for infectious disease control.

Three supply-side constraints on health system resources that apply to TB service provision in the public sector in South Africa were defined for incorporation in the model: (1) a financial constraint, characterized as the size of the available TB budget; (2) a (nonfinancial) exogenous human resources (HR) constraint, characterized as the maximum full time equivalent of nursing staff that can be employed nationally in the provision of TB services (registered, enrolled, and specialized TB nurses supply virtually all TB services in the government sector in the country); and (3) a diagnostics purchasing constraint, characterized as the maximum number of Xpert tests purchased annually by the TB program. The latter was considered as a policy constraint internal to the vertical TB budget, which is restricted by an arbitrary, a priori belief held by policymakers on the viable number of tests per TB case detected (National Department of Health, written personal communication, August 2014).

For the budget and HR constraints, 3 possible scenarios were considered: a more restrictive scenario, where resources over time are assumed to be virtually “static,” their increase uniquely driven by the underlying growth rate of gross domestic product and population, respectively; a medium scenario, where static health resources are reallocated to TB services for the period covered by the 2017-2022 NTP to match the share of disease burden caused by TB, reported at 15% by NDoH; and a least restrictive scenario, where 15% of “dynamic” health resources that realize their full growth potential is reallocated to TB over the same period. The maximum potential growth in the TB budget was informed by a fiscal space analysis, whereas the availability of nurse time for TB was estimated from routine data on historical workforce growth.^18^ One single scenario was considered for the diagnostics constraint, setting the limit to a ratio of 20 Xpert tests purchased for every TB case diagnosed in the previous budgeting period. Details on how all constraints were parameterized are published in Bozzani et al (2018).^18^

### Cost-Effectiveness Model

We used a mathematical model of TB transmission to estimate the number of TB cases, TB mortality, and the use of TB services under each of the intervention scenarios (intervention 1 being the base case and interventions 2-10 exploring different combinations of screening and diagnostic algorithms; see Fig. 1).^19^ The model was calibrated for the year 2015 and cost projections were generated for a 20-year period up to 2035 by attaching unit costs to model outputs. Unit costs for the TB case-finding and diagnostic interventions as well as for the routine TB services affected by the policy changes were constructed using ingredients costing from ongoing studies as well as from published sources (Table 1).^18^^,^^20^, ^21^, ^22^, ^23^ Only costs incurred by health service providers were considered. All costs are presented in 2016 US dollars, and a discount rate of 3% was applied to future costs.

**Table 1:** Unit costs of TB services and interventions.^18^

Intervention	Description	Unit	Unit cost of output (2016 US$)	Source
Nurse time	1 minute of professional nurses' time	Per minute	0.34	Nicola Foster, unpublished XTEND data
Inpatient day	Cost of hospitalization	Per bed-day	44.44	Edina Sinanovic, unpublished XTEND data
OPD visit	Nurse consultation, 12 minutes average duration	per event	4.08	Nicola Foster, unpublished XTEND data
IPT treatment	One OPD visit a month (at half cost as on HIV) + INH + Xpert cost every year	per month	7.81	Salome Charalambous, written personal communication, October 2016
First-line TB treatment	Facility-based observation, 2 months intensive phase; 4 months continuation phase	per patient month	21.43	Treatment regimens from The Aurum Institute (2016).^20^ Drug prices from National Department of Health's master procurement catalog,April 8, 2016.^21^ Only 20% of patients are treated under DOTS; the rest visit the facility once a month to collect drugs (Dr Lindiwe Mvusi, National Department of Health, oral personal communication, November 2016).
MDR-TB treatment	6 months intensive phase; 18 months continuation phase	per patient month	359.06	As for first-line treatment. From Sinanovic et al (2015),^22^ 40% of patients are hospitalized during intensive phase; the rest receive fully decentralized treatment
TB diagnostics	Sum of costs of first- and second-line diagnostic tests, including visits and antibiotics∗	per person diagnosed	53.65	Costs of first-line diagnostics from Cunnama et al (2016),^23^ Costs of monitoring tests from Edina Sinanovic, unpublished XTEND data
WHO symptoms screener	4 minutes of a professional nurse	per suspect screened	1.36	Nicola Foster, unpublished XTEND data
Cough triage	1.3 minutes of professional nurse asking cough question	per suspect screened	0.68	MERGE trial

DOTS indicates directly observed treatment, short course; INH, isoniazid; IPT, isoniazid preventive therapy; MDR-TB, multidrug-Resistant tuberculosis; OPD, outpatient department.

*Note.* Shaded activities represent interventions introduced or modified under the 2017-2022 National TB Plan, as opposed to routine services.

∗ Cost per person diagnosed calculated as a weighted average of the unit costs of each test from the XTEND trial, where the weights represent the probability of receiving each test experienced by diagnosed patients in the XTEND cohort.

Health benefits of the interventions were measured in terms of the disability-adjusted life years (DALYs) averted by each intervention compared to the base case. DALYs were calculated using transmission model outputs, including deaths by age and year and the annual population distribution across TB- and HIV-related health states. Disability weights were derived from a multicountry valuation,^24^ assuming (1) asymptomatic HIV (CD4 > 350) equal to “generic uncomplicated disease” (0.054); (2) those with active TB and either asymptomatic HIV or on ART experience the same disability as those who are HIV-uninfected (0.331); and (3) those with acquired immunodeficiency syndrome (AIDS, CD4 < 200) experience the same disability whether or not they have active TB (0.547). Remaining life expectancy was estimated throughout the period considered in the analysis from the South African life tables.^25^

### Estimating ICERs

The model was first run to estimate costs and effects of scaling up the interventions in the absence of constraints. The individual constraints scenarios were then applied independently to the model by reducing the intervention coverage such that the projected resource requirements remained below the constraint over the entire analytic horizon. The cost-effectiveness of the interventions was then assessed at the coverage that could be achieved within the available resources. If target coverage was not achieved, then a real constraint was identified and the model was re-run at a reduced coverage, such that the projected resource requirements remained below the constraint over the entire analytic horizon. Budget requirements were estimated by the standard economic model attaching unit costs to TB transmission model outputs. HR requirements were similarly calculated by attaching to model outputs an estimate of the nurse minutes required to deliver the TB screening and diagnostic interventions as well as the routine services along the TB care cascade.^18^ The diagnostics constraint was incorporated as a multiplier in the model, which limited intervention coverage once the set ratio of Xpert tests to TB notifications was exceeded.

For those interventions that had to be delivered at a reduced coverage under any of the HR constraint scenarios, the costs of “relaxing” the constraint by adding extra nurses to the workforce to achieve full coverage were calculated^18^ to produce a third set of ICERs alongside the unconstrained and constrained ICERs. These represent the true opportunity cost of delivering the interventions. We assumed no additional health system investment would be necessary for relaxing the financial and diagnostics constraints (besides increasing the TB budget and purchasing additional Xpert tests, respectively), so that the costs of achieving full coverage would be equal to those predicted by the model under the unconstrained scenario.

## Results

The incremental costs, DALYs averted, and ICERs compared to base case for all the interventions under the unconstrained and constrained scenarios (only for scenarios that had a realistic impact on feasibility of one or more interventions) are presented in Table 2. Detailed results on cost estimation and the effects of the constraints on intervention impact have been presented elsewhere.^18^^,^^19^

**Table 2:** Incremental costs, DALYs averted, and ICERs∗ for interventions 2 through 10 compared to intervention 1 (base case) under selected constraint scenarios.

Intervention (target coverage†)	Constraint scenario	Incremental costs (US dollars, thousands)	DALYs averted ( thousands)	Incremental cost per DALY averted (US dollars)
2 (100% Xpert coverage)	Unconstrained	334 654	299	1121
	HR (least limiting)	334 654	299	1121
	HR (medium)	334 654	299	1121
	Financial (medium)	334 654	290	1153
	Diagnostics	189 237	234	809
3 (90% follow-up of Xpert negatives)	Unconstrained	73 201	86	847
	HR (least limiting)	73 201	86	847
	HR (medium)	73 201	86	847
	Financial (medium)	73 201	86	847
	Diagnostics	68 411	85	806
4 (2 + 3)	Unconstrained	417 027	381	1093
	HR (least limiting)	417 027	381	1093
	HR (medium)	417 027	381	1093
	Financial (medium)	417 027	381	1093
	Diagnostics	268 375	318	844
5 (cough triage in 100% of patients with HIV)	Unconstrained	–496 799	34	–14 588
	HR (least limiting)	–496 799	34	–14 588
	HR (medium)	–496 799	34	–14 588
	Financial (medium)	–496 799	34	–14 588
	Diagnostics	–395 416	89	–4425
6 (cough triage in 90% PHC patients)	Unconstrained	525 977	255	2061
	HR (least limiting)	525 514	255	2060
	HR (medium)	126 528	56	2248
	Financial (medium)	525 977	255	2061
	Diagnostics	91 886	46	1989
7 (WHO screener in 100% of patients with HIV)	Unconstrained	2 693 662	649	4148
	HR (least limiting)	1 359 565	420	3241
	HR (medium)	130 455	52	2489
	Financial (medium)	2 693 662	649	4148
	Diagnostics	128 738	56	2303
8 (WHO screener in 90% PHC patients)	Unconstrained	3 800 388	907	4190
	HR (least limiting)	463 344	27	17 201
	HR (medium)	170 109	-78	–2193
	Financial (medium)	3 800 388	907	4190
	Diagnostics	–31 106	-102	304‡
9 (4 + 6)	Unconstrained	988 363	620	1595
	HR (least limiting)	987 853	619	1595
	HR (medium)	512 389	414	1237
	Financial (medium)	988 363	620	1595
	Diagnostics	335 806	345	972
10 (4 + 8)	Unconstrained	4 631 162	1,222	3789
	HR (least limiting)	932 298	401	2327
	HR (medium)	606 355	303	2004
	Financial (medium)	4 606 292	1,220	3776
	Diagnostics	123 317	212	582

DALYs indicate disability-adjusted life years; GDP, gross domestic product; HR: human resources; ICER, incremental cost-effectiveness ratio; PHC, primary healthcare.

*Note.* Dominant interventions shown on expansion path. Strongly (costlier, less effective than another individual intervention) and weakly (costlier, less effective than a combination of nonmutually exclusive interventions) dominated interventions shown in lighter shade.

∗ Cumulative values for 20-year analytic horizon (2016-2035) discounted at 3% per year. All costs reported in 2016 US dollars.

† Target coverage refers to the coverage achievable under the unconstrained scenario.

‡ Scenario produces negative costs and health benefits compared to base case. Reported ICER represents the costs and effects of moving from scenario to base case, as opposed to the other comparisons.

### Cost-Effectiveness Ranking Without Constraints

**Figure 2:**
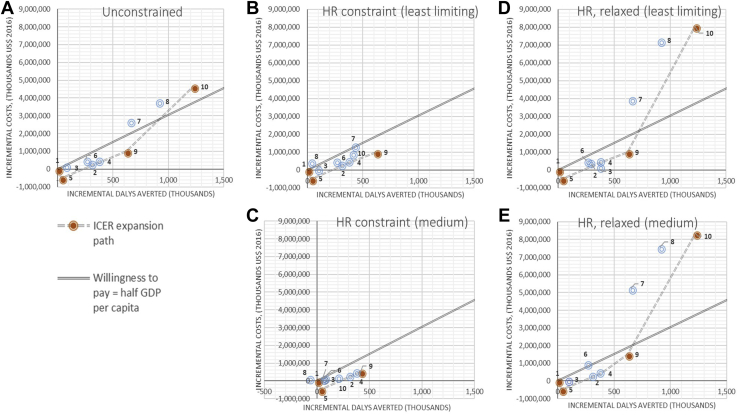
Cost-effectiveness planes for selected constraints scenarios. (A) Unconstrained. (B) HR constraint (least limiting). (C) HR constraint (medium). (D) HR, relaxed (least limiting). (E) HR, relaxed (medium).

Figure 2 presents the ICER ranking of intervention options on the cost-effectiveness plane. Dominant options are shown on the expansion path from intervention 1 (the base case) at the origin. Interventions that are strongly (costlier and less effective than an individual intervention) or weakly dominated (costlier and less effective than a combination of nonmutually exclusive interventions) are shown outside the expansion path. When supply-side constraints are not taken into consideration (Fig. 2, panel A), the option with the highest costs and the most DALYs averted compared to the base case was intervention 10, the combination of strengthening the diagnostic algorithm and screening 90% of all patients in primary healthcare (PHC) using the WHO tool. Assuming an indicative willingness-to-pay threshold equal to half the gross domestic product per capita per DALY averted ($3044), South Africa would adopt intervention 9 under this scenario, combining the strengthening of the Xpert algorithm with use of the less sensitive cough triage in the PHC population. This intervention would require a twofold increase in the total TB budget for South Africa for 2015.

### Cost-Effectiveness Ranking With Constraints

The least limiting financial constraint was not exceeded by any of the interventions, although the most limiting financial and HR constraints were exceeded by almost all interventions at some point during the analytical time horizon, indicating that any TB case-finding policy change would not be feasible in South Africa without some reprioritization of funds. The medium financial constraint would cause a shortfall of approximately $25 million over the period 2016-2035 and thus reduce overall intervention effectiveness (Table 2).

Incorporating nonfinancial constraints influences the ranking of interventions under the least restrictive and medium HR constraints (Fig. 2, panels B-C), as well as under the diagnostic constraint. In these 3 scenarios, intervention 10 was (weakly) dominated by intervention 9 (combination of strengthened diagnostic algorithm and cough triage for screening PHC patients), which became more effective than option 10 at reduced coverage and had a lower ICER. The least restrictive and medium HR constraints had a substantial impact on the coverage of all ICF interventions (6-10), except for the use of cough-based screening in HIV clinics (5), which constitutes a reversal of the current guidelines recommending use of the WHO tool among patients with HIV, adhered to in approximately 40% of cases.^26^ Overall, the diagnostics constraint caused the greatest reductions in the impact of all intervention options involving the strengthening of the diagnostic algorithm (interventions 2-4) as well as the expansion of ICF to all PHC patients using any screening tool (interventions 6, 8, 9, and 10), due to the limit it placed on the consequential scaling up of Xpert.

### Cost-Effectiveness of Relaxing HR Constraints

Figure 2, Panels B2-C2 show the ICERs considering the health system investment for training, hiring, and deploying the additional nurses required to deliver the interventions at the target coverage. Once the constraints were relaxed, option 10 once again displayed the highest ICER, as in the unconstrained scenario (Fig. 2, panel A). Investing in the generation of extra HR capacity to deliver the strengthened diagnostic and WHO screening algorithm intervention at the desired coverage requires an increase of approximately 60% in the TB service delivery budget during the 2016-2035 period compared with the current expenditure level (from about $6.3 billion to $10 billion), and these additional costs substantially decrease cost-effectiveness compared with the threshold.

## Discussion

We demonstrate an empirical approach to incorporating nonfinancial constraints into cost-effectiveness analysis that can inform investment decisions where health sectors face resource limitations or policy restrictions. In our case study, we illustrated to decision makers the consequences of not addressing constraints (reduced intervention impact) and the returns on investment in removing them (costs of relaxing HR constraints). As such, we present an approach that reflects an understanding of health sectors facing complex short-run constraints and that allows policy makers to explore combinations of investments and to optimize between the short and long run.

The prior beliefs of policy makers around the potential lack of feasibility of expanding HR-intensive ICF strategies that generate high volumes of Xpert diagnostic tests further down the TB cascade in an overstretched health system were reinforced by our analysis. Critically, we find that, in the presence of HR constraints, expanding the TB budget is not enough to achieve the desired coverage target of ICF interventions. This highlights the importance of long-term investments in training and hiring more nurses, as well as the presence of a time lag for deploying this extra workforce, which in turn may impact intervention effect. Our findings are in line with van Baal and colleagues, whose theoretical model illustrated that, in the short term, choosing interventions whose feasibility is more dependent on the availability of physical inputs such as HR has a higher opportunity cost than choosing less resource-intensive options.^1^ Adding to van Baal’s approach, the present work presents a possibility for dealing with available routine data that are also independent of specific opportunity cost thresholds. Moreover, it expands the choice set by presenting the option of relaxing health system constraints and, although not shown in the case study presented, could accommodate an analysis of returns to scale for physical inputs.

Despite being feasible, our approach has some potential limitations and requires further development. First, although we completed the analysis within a policy-defined time frame, it required considerable additional effort compared to conventional approaches. In particular, we had to obtain high-quality published and unpublished cost data from previous studies in South Africa, we gained access to and collated detailed data on HR supply, and we spent considerable time defining constraints as well as discussing and deliberating with decision makers (TB Think Tank) on how to represent them in the model. Our approach may thus be less feasible where data availability and formal planning structures are more limited. Second, our conclusions rely on the assumption that, in the presence of constraints, existing TB services would not be scaled back to accommodate new screening interventions. Although rare in practice, decision makers might be willing to consider divesting from existing activities to increase coverage of desirable new interventions. Moreover, we have assumed that there would be no interactions between the constraints, as they applied to different types of resources. But because constrained inputs are all funded from the health budget, interactions might occur, and thus the analysis would need to apply the constraints to the model simultaneously and quantify the extent of the interactions. Finally, we have considered a single change in ICF coverage of screening in each scenario over time, without acknowledging that policies are dynamic and responsive to system changes.

## Conclusion

Despite its limitations, the approach presented earlier emphasizes the importance and feasibility of supplying decision makers with information on the “real-world” cost-effectiveness and performance of intervention options under different types of health system constraints. In many LMICs, decisions on the adoption of new technologies are coupled with scale-up decisions and are not seen as separate processes. The further testing of approaches such as the one presented here is required to ensure that health sector decision makers can explore the optimal balance between short-run purchasing of new technologies and long-term investment to reduce nonfinancial health systems constraints.

## Article and Author Information

**Accepted for Publication:** May 9, 2020

**Published Online:**

doi: https://doi.org/10.1016/j.jval.2020.05.021
